# U-shaped association between sleep duration and frailty in Chinese older adults: a cross-sectional study

**DOI:** 10.3389/fpubh.2024.1464734

**Published:** 2025-01-07

**Authors:** Yanliqing Song, Haoqiang Liu, Kenan Gu, Yue Liu

**Affiliations:** ^1^College of Sports, Nanjing Tech University, Nanjing, China; ^2^School of Athletic Performance, Shanghai University of Sport, Shanghai, China

**Keywords:** frailty, China, sleep duration, older adults, COVID-19

## Abstract

**Objective:**

As the population ages, understanding the association between sleep patterns and physical frailty in older adults is crucial for formulating effective health interventions. This study aimed to explore the relationship among nap time, nighttime sleep duration, and physical frailty in older Chinese individuals; establish recommended sleep times; and provide a scientific and reasonable basis for the prevention and management of frailty in older adults.

**Methods:**

On the basis of the 2020 China Health and Retirement Longitudinal Study database, demographic information, health data, and lifestyle information of the research subjects were obtained. A total of 5,761 survey participants were included, and logistic regression and restricted cubic splines were used to explore the association between sleep duration and frailty.

**Results:**

In our cross-sectional analysis, the duration of napping in older adults did not show a significant correlation with frailty. The optimal nighttime sleep interval for older adults was 7–8 h, and the maximum health benefit was achieved when nighttime sleep reached 7.5 h. Compared with older adults in China who slept 6–8 h at night, those with a sleep duration of <6 h (OR = 1.58, 95% CI: 1.36–1.82) were more likely to be frail. After adjusting for all covariates such as smoking, multimorbidity, self-rated health, social events, education level, and frequency of physical activity, we found no interaction between gender and age concerning sleep duration.

**Conclusion:**

The potential correlation between nighttime sleep duration and frailty in older adults is basically U-shaped. Older Chinese adults with a moderate nighttime sleep duration of 7–8 h exhibited the lowest likelihood of frailty than their counterparts. The duration of napping is not related to the likelihood of frailty in older people. Thus, the importance of sufficient nighttime sleep for the health of older adults must be emphasized.

## Introduction

Physical frailty is a transitional state between robust health and disability, marked by fatigue, reduced muscle mass and strength, and other decreases in physiological functions (1). This is a reversible dynamic pathological process that poses a major challenge for healthy aging (2). The global prevalence of frailty ranges from 4.0 to 59.1%, whereas that in China is 7.0–28.0%, indicating an annual upward tendency (3). Frailty can result in the loss of balance ability in the body and increase the risk of illness, disability, and mortality (4). Adverse outcomes related to frailty include falls, fractures, functional deterioration, and admission to nursing facilities (5). However, frailty can be reversed if treated properly. Targeted therapy of potential causes is crucial to prevent frail older adults from developing more serious diseases (6).

Sleep is essential for health, facilitating hormone secretion, energy metabolism, glucose and cardiovascular regulation, as well as self-regulation and the recovery of physiological processes (7). Recent studies have linked sleep with frailty, including sleep complaints, insufficient or excessive sleep, insomnia, and poor sleep quality (8, 9). With advancing age, changes in sleep duration and the prevalence of sleep disorders are common among older adults, and such individuals with long sleep durations often have poor sleep quality (10). Napping is common among older adults because of various factors including chronic diseases, lifestyle changes, cultural beliefs, and age (11). Studies have shown a significant association between poor sleep quality and frailty in older adults. Poor sleep quality may lead to decreased physical recovery ability, increasing the risk of frailty (12). Excessive and insufficient sleep duration may be related to frailty. Excessive sleep duration may indicate underlying health issues, while inadequate sleep duration may lead to a decline in physical function (13). Napping is prevalent among older adults, and research indicates that long naps are associated with several adverse outcomes in older adults, primarily including depressive symptoms and diabetes (14, 15). About 20–60% of older adults nap during the day worldwide (16). Although previous studies have identified a relationship between sleep duration and physical frailty, most research focuses on nighttime sleep duration (17, 18). Only a few studies have paid attention to the correlation between midday sleep duration and cognitive frailty (19). However, the data from these studies were all obtained before the onset of COVID-19.

The COVID-19 pandemic has significantly influenced society, altering individuals’ lifestyle and health. Consequently, nations have implemented lockdowns and social distancing measures to mitigate the transmission of the virus. These measures have disrupted individuals’ daily routines, including physical exercise and sleep patterns, leading to feelings of loneliness, depression, and sleep problems, particularly affecting older adults (20). These policies restrict outdoor activities for older adults, resulting in a decline in physical function, which is characterized by reduced muscle mass, decreased mobility, and increased risk of non-communicable diseases (21). During the 2019 coronavirus disease pandemic, a relationship between nighttime sleep and frailty was reported among older adults in Thailand, establishing that poor sleep quality and difficulty falling asleep are risk factors for frailty (22). Therefore, the COVID-19 virus may intensify the incidence of frailty in older adults. Amid the COVID-19 restrictions in China, there is insufficient evidence showing an association between physical frailty in older adults and sleep duration. In the current work, we analyzed the latest China Health and Retirement Longitudinal Study (CHARLS) data obtained during the COVID-19 pandemic. We selected this dataset because it best matched the frailty characteristics of older adults at this stage.

This study aimed to explore the relationship among nap time, nighttime sleep duration, and frailty in older adults, as well as establish the optimal sleep duration. This work aimed to provide a scientific and reasonable basis for the prevention and management of frailty in residents. By adjusting nap and nighttime sleep periods, the physical function and quality of life in older adults can be effectively improved. The study findings can provide a basis for the development of public health policies, assisting governments and relevant organizations in creating health interventions aimed at older adults, thereby reducing the burden of frailty on society and the healthcare system. Additionally, the findings can guide community and family care, assisting caregivers and relatives in enhancing their support for frail older adults by providing personalized care plans, fostering healthy aging in this demographic.

### Organization of the manuscript

**Section 1** serves as the introduction, outlining the background and significance of the study.

**Section 2** describes the methods used in the study, including data collection and statistical analysis.

**Section 3** presents the results of the analysis, emphasizing key findings.

**Section 4** discusses the implications of the findings, compares them with previous studies, and outlines limitations.

**Section 5** concludes the study by summarizing the principal findings and their relevance to the field.

## Methods

### Study population

The data for this study were derived from the 2020 CHARLS data. CHARLS is the first nationally representative survey of middle-aged and older individuals in China. It uses a multi-stage PPS random sampling method based on implicit stratification to guarantee the sample’s representativeness (23). Specifically, in the PPS sampling method, primary sampling units (PSUs) are selected based on population size, with probabilities proportional to their size. Subsequently, secondary sampling units (households) are randomly chosen within each PSU. This approach effectively improves the sample’s representativeness and reduces bias. The CHARLS questionnaire offers data from various modules, including sociodemographic information, psychological status, and health status. Data from each module were gathered via in-person interviews to guarantee accuracy and completeness. The survey included standardized questionnaire tools, and investigators received extensive training to ensure consistency and reliability in the data collection process. The Biomedical Ethics Committee of Peking University granted approval for the 2020 CHARLS survey, with the approval number being IRB00001052-11015.1.2. Participants in the field survey who consented to participate were required to sign two informed consent forms. One of these forms was kept by the participant, while the other was stored in the CHARLS office. The criteria for inclusion in the study were as follows: (1) individuals aged 60 years or older and (2) possessing data concerning the presence or absence of an explanatory variable. The exclusion criteria were as follows: (1) individuals younger than 60 years and (2) those with more than 10% missing values in the relevant variables. Missing values (under 10%) were replaced via multiple imputation. The study comprised 5,761 survey participants. This sample size provides sufficient statistical power to identify the association between sleep duration and frailty in older adults.

### Outcome

The duration of nighttime sleep was evaluated using the following question: “How many hours did you actually sleep each night in the past month? (The average time per night, which may be shorter than the time you spend in bed),” which represents the average time of a typical workday and rest day. Previous research on sleep duration has revealed the advantages of 6–8 h of sleep (24). A meta-analysis of 35 sleep studies classified research participants into three categories of nighttime sleep duration: short (<6 h), moderate (6–8 h), and long sleep (>8 h) (25). Nap time was assessed using the following question: “How long did you usually nap in the past month?” Researchers were divided into four groups: no nap (0 min), short nap (<30 min), moderate nap (30–90 min), and long nap (>90 min) (26).

### Explanatory variable

The frailty index evaluates the frailty status of older adults. This study refers to the frailty index standard established by Samuel (27), with the included variable standards as follows. (1) The included indicators must be controllable health-related influencing factors. For example, uncontrollable factors such as the emergence of white hair with advancing age were excluded. (2) The incidence of indicators must increase with age. (3) The indicator cannot reach a saturation state prematurely with advancing age; for instance, presbyopia, which reaches saturation at the age of 55 in older adults, cannot serve as a valid impact indicator of frailty. (4) The frailty index adheres to the principle of systematicity and must consist of indicators from several dimensions.

The frailty index standard constructed by Samuel suggests that at least 30–40 indicators should be included. When the number of included indicators is very small (about 10 or fewer), the results will also become unreliable. In other words, the inclusion of additional indicators in the frailty index enhances the accuracy of its calculation. A frailty index derived from 30 to 40 related indicators has been proven by previous studies to possess sufficient accuracy for prediction (28). In addition, research shows that the frailty index can be developed using the majority of the readily available survey indicators in health surveys (29).

According to the frailty index standard constructed by Samuel, the controllable risk factors influencing the frailty of older adults include socioeconomic status, unhealthy lifestyle, disease, dietary behavior, psychological depression, sleep disorders, and cognitive impairment. A total of 34 indicators were selected to construct the FI framework, as follows:

1. Disease: 13 kinds of chronic diseases, 2 kinds of disabilities, 3 types of visual and auditory conditions, and 2 kinds of health evaluations. 13 chronic diseases: hypertension, dyslipidemia (hyperlipidemia or hypolipidemia), diabetes or hyperglycemia, cancer and other malignant tumors, chronic pulmonary disease, liver disease, heart disease, stroke, kidney disease, gastric disease or digestive system disease, memory-related disease, arthritis or rheumatism, and asthma. 2 kinds of disabilities: physical disability and brain damage intellectual defect. 3 types of visual and auditory conditions: blindness or partial blindness, deafness or partial deafness, and dumb or severe stuttering. 2 kinds of health evaluations: self-rated health and body pain.
2. Disability: The Katz–ADL score and Lawton–IADL score include 11 indicators, which are used to assess independent living ability and instrumental daily living function. These include dressing independently, bathing independently, eating independently, moving independently (getting up and going to bed), using the toilet independently, controlling bowel and bladder function, handling housework, preparing meals, shopping, managing finances, and making phone calls (30).
3. Depression: The CESD10 scale is used for evaluation (31, 32).
4. Cognition: Cognitive function is measured using the method used in the Health and Retirement Study in the United States (33). The Telephone Interview for Cognitive Status is used to determine orientation and calculation, with target items including year, month, day, day of the week, and current season. The total score for the orientation dimension is 5 points, with each item allocated 1 point. In terms of calculation, participants were instructed to repeatedly subtract 7 from 100, performing this procedure five times, with 1 point awarded for each successful operation. Each participant was presented with ten randomly selected words, and immediate word recall was assessed by counting the number of words recalled immediately. In addition, after the participant completed the depression scale survey, calculation tests, and drawing tests, delayed word recall was assessed. The total memory score, that is, the sum of the scores for immediate and delayed word recall, totaled 20 points, with each word valued at 1 point. Here, the interviewer showed a picture to test the participant’s ability to draw it appropriately; if correct, the subject received 1 point. The total cognitive score was defined as the sum of the scores for orientation (5 points), calculation (5 points), memory (20 points), and drawing (1 point), resulting in a total score of 31. To date, there is no consensus on the diagnostic criteria for MCI (34).

Therefore, in this study, we defined MCI as age-related cognitive decline (AACD; i.e., at least 1 standard deviation (SD) below the age standard). All participants aged 60 and above were divided into groups at 5-year intervals, and participants in each age group who satisfied the AACD standard were classified as MCI. The variables of the subsequent dimensions were evaluated based on their type, with the values of two categorical variables assigned as 0 and 1, and the values of three categorical variables were assigned as 0, 0.5, and 1, respectively, and so on.

The calculation method for the FI is the number of health defects divided by the total number of subjects (34 in this study). Its range was 0–1; a high value implies high frailty, with FI ≥0.25 defined as frail.

### Other covariates

The analysis included a set of potential confounding variables. For sociodemographic data, we selected variables including age (60–69 years, 70–79 years, and 80 years and above), gender (female or male), education level (illiterate, primary school, or junior high school and above), region (urban or rural), marital status (married, divorced, widowed, or unmarried), occupational status (retired, continue to work after retirement, working, or unemployed), and living alone (yes or no). Lifestyle factors included smoking (yes or no), drinking (yes or no), social events (yes or no), and frequency of physical activity (0 times a week, 1–4 times a week, or more than 4 times a week). Health status involved multimorbidity (yes or no).

### Statistical methods

Statistical analysis was performed using R 4.2 software. The mean and standard deviation (mean ± SD) of continuous variables and the frequency (N) and percentage (%) of categorical variables were used to describe the baseline characteristics of different types of sleep duration in older adults. For continuous variables (e.g., hours of sleep), we calculated the mean and standard deviation to capture the central tendency and variability. For categorical variables (e.g., gender), we used frequency and percentage to describe the distribution. Univariate analysis was performed to test continuous variables with ANOVA and categorical variables with the *χ*^2^ test, with nap and nighttime sleep duration as the reference group to construct a logistic regression model. ANOVA was chosen for continuous variables to assess the mean differences among groups, whereas *χ*^2^ tests were used for categorical variables to evaluate the association between categorical data. The variance inflation factor (VIF) was used to measure multicollinearity among independent variables, and the Box–Tidwell method was used to test the linear relationship between the logarithmic values of continuous independent and dependent variables. Model 1 included the nighttime and nap time of older adults, Model 2 controlled for demographic variables, and Model 3 included all covariates. Restricted cubic splines were plotted to explore the dose–response relationship between sleep duration and frailty in older adults. This technique allowed us to model and visualize the nonlinear relationship between sleep duration and frailty risk, providing a nuanced understanding of the dose–response relationship. In addition, subgroup analysis revealed that, after adjusting for related covariates, age- and gender-specific factors influencing the impact of sleep duration on frailty in older adults were determined. Subgroup analyses were conducted to assess whether the relationship between sleep duration and frailty varies by age and gender, providing insights into potential effect modifiers. *p* values were used for two-sided tests, and *p* < 0.05 was considered statistically significant.

## Results

### Participant characteristics

The final effective sample of this study comprised 6,430 older adults people: 3190 were females (55.37%), with an average age of 68.27 ± 6.18 years, and 1,408 (24.44%) suffered from physical frailty. There were 3,589 people (62.30%) who were illiterate, 532 people (9.23%) residing in urban areas, 4,412 individuals (76.58%) who were married, 492 people (8.54%) reporting extremely poor self-rated health, 3,330 people (57.82%) who have not participated in any social events within the past month, 2,468 people (42.84%) with a nighttime sleep duration of less than 6 h, and 2,154 people (37.39%) without a habit of napping. The differences among the variables of region, living alone, naptime, and drinking were not statistically significant (*p* > 0.05), whereas the differences among other variables were statistically significant (*p* < 0.001). Table 1 shows specific basic characteristics.

**Table 1:** Basic characteristics of study participants (*n* = 6,430).

Variables	Total (*n* = 5,761)	Robust (*n* = 4,351)	Frailty (*n* = 1,408)	Statistic	*P*
**Age**, Mean ± SD	68.27 ± 6.18	67.97 ± 6.13	69.18 ± 6.28	*t* = −6.33	<0.001
**FI**, Mean ± SD	0.25 ± 1.04	0.17 ± 0.04	0.48 ± 2.09	*t* = −5.59	<0.001
**Gender**, *n* (%)				*χ*^2^ = 4.56	0.033
Female	3,190 (55.37)	2,445 (56.17)	745 (52.91)		
Male	2,571 (44.63)	1908 (43.83)	663 (47.09)		
**Region**, *n* (%)				*χ*^2^ = 0.40	0.527
City	532 (9.23)	396 (9.10)	136 (9.66)		
Countryside	5,229 (90.77)	3,957 (90.90)	1,272 (90.34)		
**Marital status**, n (%)				*χ*^2^ = 3.85	0.278
Married	4,412 (76.58)	3,359 (77.17)	1,053 (74.79)		
Divorce	62 (1.08)	48 (1.10)	14 (0.99)		
Widowed	1,257 (21.82)	924 (21.23)	333 (23.65)		
Unmarried	30 (0.52)	22 (0.51)	8 (0.57)		
**Living alone**, *n* (%)				*χ*^2^ = 0.09	0.763
No	5,192 (90.12)	3,926 (90.19)	1,266 (89.91)		
Yes	569 (9.88)	427 (9.81)	142 (10.09)		
**Self-rated health**, *n* (%)				*χ*^2^ = 390.04	<0.001
Very bad	492 (8.54)	255 (5.86)	237 (16.83)		
Bad	1,322 (22.95)	844 (19.39)	478 (33.95)		
Fair	2,796 (48.53)	2,229 (51.21)	567 (40.27)		
Good	581 (10.09)	507 (11.65)	74 (5.26)		
Very good	570 (9.89)	518 (11.90)	52 (3.69)		
**Naptime**, *n* (%)				*χ*^2^ = 1.01	0.798
0 min	2,154 (37.39)	1,641 (37.70)	513 (36.43)		
<30 min	826 (14.34)	621 (14.27)	205 (14.56)		
30–90 min	1,572 (27.29)	1,188 (27.29)	384 (27.27)		
>90 min	1,209 (20.99)	903 (20.74)	306 (21.73)		
**Nighttime sleep**, *n* (%)				*χ*^2^ = 109.20	<0.001
< 6 h	2,468 (42.84)	1703 (39.12)	765 (54.33)		
					
6–8 h	2,750 (47.73)	2,240 (51.46)	510 (36.22)		
>8 h	543 (9.43)	410 (9.42)	133 (9.45)		
**Social events**, *n* (%)				*χ*^2^ = 97.27	<0.001
No	3,330 (57.82)	2,357 (54.17)	973 (69.11)		
Yes	2,429 (42.18)	1994 (45.83)	435 (30.89)		
**Smoking**, *n* (%)				*χ*^2^ = 34.17	<0.001
No	3,467 (60.18)	2,713 (62.32)	754 (53.55)		
Yes	2,294 (39.82)	1,640 (37.68)	654 (46.45)		
**Drinking**, *n* (%)				*χ*^2^ = 11.14	<0.001
No	3,977 (69.06)	3,055 (70.21)	922 (65.48)		
Yes	1782 (30.94)	1,296 (29.79)	486 (34.52)		
**Education level,** *n* (%)				*χ*^2^ = 21.10	<0.001
Illiterate	3,589 (62.30)	2,649 (60.85)	940 (66.76)		
Elementary school	1,240 (21.52)	950 (21.82)	290 (20.60)		
Junior high school and above	932 (16.18)	754 (17.32)	178 (12.64)		
**Multimorbidity**, *n* (%)				*χ*^2^ = 529.42	<0.001
No	1,446 (25.10)	1,418 (32.58)	28 (1.99)		
Yes	4,315 (74.90)	2,935 (67.42)	1,380 (98.01)		
**Frequency of physical activity**, *n* (%)				*χ*^2^ = 78.81	<0.001
0 times a week	796 (13.82)	504 (11.58)	292 (20.74)		
1–4 times a week	827 (14.36)	619 (14.22)	208 (14.77)		
more than 4 times a week	4,138 (71.83)	3,230 (74.20)	908 (64.49)		
**Age,** *n* (%)				*χ*^2^ = 49.02	<0.001
60–69	3,699 (64.21)	2,904 (66.71)	795 (56.46)		
70–79	1724 (29.93)	1,207 (27.73)	517 (36.72)		
80 and above	338 (5.87)	242 (5.56)	96 (6.82)		
**Occupational status**, *n* (%)				*χ*^2^ = 110.15	<0.001
Retired	45 (0.78)	36 (0.83)	9 (0.64)		
Continue to work after retirement	85 (1.48)	70 (1.61)	15 (1.07)		
Working	3,765 (65.35)	2,997 (68.85)	768 (54.55)		
Unemployed	1866 (32.39)	1,250 (28.72)	616 (43.75)		

*t*, *t*-test; *χ*^2^ (df), Chi-square test.

SD, standard deviation.

### Logistic regression of the relationship between sleep duration and frailty in older adults

For the diagnosis of multicollinearity, a VIF value greater than 10 is considered indicative of multicollinearity and will be eliminated. In our data, all VIF values were below 10, so no variables needed to be excluded. The Box–Tidwell test results showed that the continuous independent variables (age, nighttime sleep, and nap sleep time) had a linear relationship with the dependent variable (frailty) (*p* > 0.05). The results of the logistic regression model showed that, after controlling for all covariates in Model 3, older adults with nighttime sleep durations of 6–8 h and over 8 h were less likely to experience frailty compared with those with less than 6 h of nighttime sleep. The duration of napping in older adults did not show a significant correlation with frailty (Table 2).

**Table 2:** Logistic regression table of the relationship between sleep duration and frailty in older adults.

Variable	Model one	Model two	Model three
Nighttime sleep			
< 6 h	1.00 (Reference)	1.00 (Reference)	1.00 (Reference)
6–8 h	0.50 (0.44 ~ 0.57) **	0.51 (0.45 ~ 0.59) **	0.63 (0.54 ~ 0.73) **
>8 h	0.71 (0.57 ~ 0.87) **	0.66 (0.53 ~ 0.82) **	0.71 (0.56 ~ 0.90) *
Nap time			
0 min	1.00 (Reference)	1.00 (Reference)	1.00 (Reference)
<30 min	1.09 (0.90 ~ 1.31)	1.11 (0.92 ~ 1.35)	1.08 (0.88 ~ 1.34)
30–90 min	1.12 (0.96 ~ 1.31)	1.09 (0.93 ~ 1.27)	1.07 (0.90 ~ 1.27)
>90 min	1.19 (1.01 ~ 1.41) *	1.11 (0.94 ~ 1.32)	1.08 (0.90 ~ 1.31)

Model 1 includes nighttime and nap time of older adults, model 2 controls for demographic variables, and model 3 has all covariates.

### Dose–response relationship between sleep duration and frailty in older adults in China

**Figure 1:**
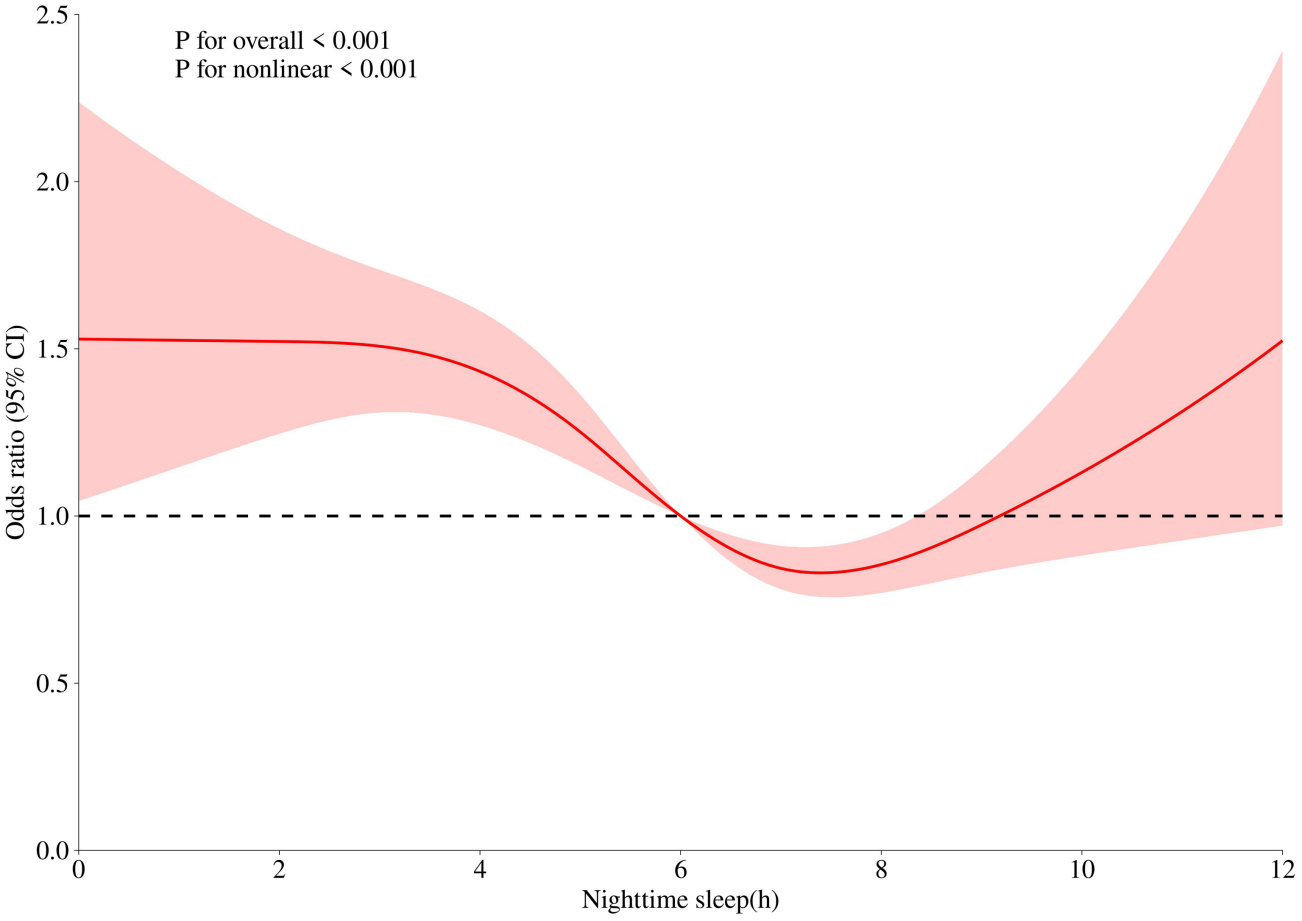
Dose-response relationship between nighttime sleep duration and frailty in the older adults. The x-axis represents sleep duration. The y-axis indicates the OR calculated by the model. The shadowed area represents the 95% confidence interval (test for overall trend: *P* < 0.001; test for nonlinear trend: *P* < 0.001).

The dose–response relationship graph depicting sleep duration and frailty among older adults in China reveals a U-shaped relationship (*p* < 0.001), indicating that frailty in older adults in China is minimized at a nighttime sleep duration of 7–8 h. Figure 1 presents details of this graph.

Given that frailty among older adults in China is lower level with a nighttime sleep duration of 7–8 h, we used 6–8 h as the control group to construct a logistic regression model. The results are shown in Table 3. Compared with older adults in China who sleep 6–8 h at night, a sleep duration of <6 h (OR = 1.58, 95% CI: 1.36–1.82) is associated with an increased probability of frailty.

**Table 3:** Logistic regression to adjust the association between nighttime sleep grouping and frailty in older adults.

Variable	Beta	S.E	Z	P	OR (95%CI)
Nighttime sleep time(h)					
6-8 h					1.00 (Reference)
< 6 h	0.45	0.07	6.11	<0.001	1.58 (1.36 ~ 1.82)
>8 h	0.12	0.13	0.95	0.345	1.13 (0.88 ~ 1.44)

The model was adjusted for all covariates.

### Subgroup analysis of nighttime sleep duration and frailty in older adults

After adjusting for all covariates such as smoking, multimorbidity, self-rated health, social events, education level, and frequency of physical activity, this study identified age- and gender-specific factors influencing nighttime sleep duration on frailty in older adults.

For all nighttime sleep durations in older adults, we found no significant interaction between gender and frailty (*p* > 0.05). This result indicated that the effect of nighttime sleep duration on frailty in older male and female adults did not differ. Within a nighttime sleep duration of <6 h, both males and females exhibited a significantly increased risk of frailty. Within a nighttime sleep duration of 6–8 h, the likelihood of frailty significantly decreased for males and females. Within a nighttime sleep duration of >8 h, the effect of gender on frailty was not statistically significant, indicating that a sleep duration exceeding 8 h may not significantly affect frailty in older adults (Table 4).

**Table 4:** Subgroup analysis of age and gender-specific factors for nighttime sleep duration and frailty in older adults.

Variables	*n* (%)	Robust	Frailty	OR (95%CI)	*P*	*P* for interaction
Nighttime sleep<6 h						
Gender						0.816
Female	3,190 (55.37)	285/1637	460/1553	1.54 (1.28 ~ 1.86)	<0.001	
Male	2,571 (44.63)	358/1656	305/915	1.58 (1.28 ~ 1.93)	<0.001	
Age						0.392
60–69	3,699 (64.21)	391/2197	404/1502	1.42 (1.19 ~ 1.70)	<0.001	
70–79	1724 (29.93)	216/930	301/794	1.76 (1.39 ~ 2.22)	<0.001	
80 and above	338 (5.87)	36/166	60/172	1.69 (0.94 ~ 3.04)	0.077	
Nighttime sleep 6-8 h						
Gender						0.878
Female	3,190 (55.37)	519/1831	226/1359	0.66 (0.54 ~ 0.80)	<0.001	
Male	2,571 (44.63)	379/1180	284/1391	0.67 (0.55 ~ 0.82)	<0.001	
Age						0.480
60–69	3,699 (64.21)	469/1799	326/1900	0.73 (0.61 ~ 0.87)	<0.001	
70–79	1724 (29.93)	356/995	161/729	0.61 (0.48 ~ 0.77)	<0.001	
80 and above	338 (5.87)	73/217	23/121	0.62 (0.33 ~ 1.16)	0.134	
Nighttime sleep>8 h						
Gender						0.977
Female	3,190 (55.37)	686/2912	59/278	0.88 (0.63 ~ 1.23)	0.465	
Male	2,571 (44.63)	589/2306	74/265	0.91 (0.66 ~ 1.26)	0.561	
Age						0.903
60–69	3,699 (64.21)	730/3402	65/297	0.90 (0.65 ~ 1.25)	0.534	
70–79	1724 (29.93)	462/1523	55/201	0.82 (0.57 ~ 1.17)	0.272	
80 and above	338 (5.87)	83/293	13/45	0.79 (0.34 ~ 1.85)	0.591	

OR, odds ratio, CI, confidence interval.

No significant interaction was found between age and the effect of nighttime sleep duration on frailty in older adults (*p* > 0.05). However, the likelihood of frailty varies across different age cohorts. Older adults under 80 years of age exhibited a higher propensity for frailty associated with age while obtaining <6 h of nighttime sleep, and those experiencing insufficient sleep demonstrated an elevated risk of frailty. Adults under 80 years of age were less prone to frailty with 6–8 h of nighttime sleep. The effect of age on the probability of frailty was not statistically significant for a nighttime sleep duration of >8 h.

## Discussion

### Major findings

This cross-sectional analysis revealed a U-shaped association between the duration of nighttime sleep and the likelihood of frailty. The length of midday sleep is linearly correlated with the occurrence of frailty. The ideal nighttime sleep duration for older adults is 7–8 h, with maximum health benefits achieved at 7.5 h of sleep. Older adults in China who sleep less than 6 h are more likely to be frail compared with those who sleep 6–8 h (OR = 1.58, 95% CI: 1.36–1.82). The duration of napping in older adults is not significantly associated with the likelihood of frailty. After adjusting for all covariates such as smoking, multimorbidity, self-rated health, social events, education level, and frequency of physical activity, subgroup analysis showed no relationship between nap time and the frailty status of older adults. By contrast, we observed a relationship between nighttime sleep time and the frailty status of older adults, which was consistent across genders and among individuals under 80 years of age. The likelihood of frailty increases for older persons under 80 years of age who have less than 6 h of sleep, but 6–8 h of sleep appears to be associated with a lowered incidence of frailty. These findings underscore the importance of adequate nighttime sleep for the health of older adults.

### Comparison with previous study

Napping is one of the main forms of daytime sleep. Previous research have demonstrated a relationship between daytime sleep duration and adverse health outcomes (35, 36). The duration of napping in older adults is not significantly associated with the likelihood of frailty. This contradicts the results of a previous study, which indicated that extended napping times are associated with an increased probability of cognitive and physical frailty among older adults in nursing facilities (37). Prior longitudinal research on factors related to cognitive frailty in middle-aged and older adults based on the ecological model of health identified a nap time of 30–60 min as a protective factor (38). However, this study also determined that an appropriate nap time was not associated with physical frailty in older adults.

The duration of nighttime sleep and the likelihood of frailty exhibit a U-shaped relationship (39, 40). Short and long sleep durations are associated with health issues, whereas adequate high-quality sleep promotes the body’s self-repair mechanisms (41). Research indicates that the odds ratio (OR) for physical weakness is significantly higher for nighttime sleep durations of ≤6 h (OR = 1.53, 95% CI: 1.26–1.87) and ≥ 9 h (OR = 2.39, 95% CI: 1.90–3.00) compared with 6.1–8.9 h (41). A meta-analysis of 10 studies showed that nighttime sleep durations of >8 h and < 6 h are significantly associated with a heightened risk of frailty in older adults compared with a nighttime sleep duration of 6–8 h (42). Patients with short (<6 h) and long (≥9 h) sleep durations have FI scores that are 16% (95% CI 6, 28%) and 11% (95% CI 0, 23%) higher, respectively (43). The observational results regarding the relationship between sleep duration and frailty are also inconsistent. Previous studies involving Japanese (41) and Danish (44) populations indicated that long (≥9 h) and short (≤6 h or ≤ 5 h) sleep durations are associated with an increased likelihood of frailty. However, in older adults in the United States (39), only extended sleep durations (≥10 h, ≥9 h, or ≥ 8 h) are associated with an increased incidence of frailty.

This study found that the likelihood of frailty increases for older adults who sleep less than 6 h; however, when nighttime sleep exceeds 8 h, this relationship does not vary among different gender groups. The conclusions of previous studies are inconsistent. One study found that only a short sleep duration (<5 h or < 6 h) is related to frailty in women, but not in men (45). A survey of older adults in Korea indicated that, after adjusting for confounding variables, the OR value for frailty in women is significantly higher in the short sleep duration group (OR = 2.61, 95% CI = 1.91–4.83) and the long sleep duration group (OR = 2.57, 95% CI = 1.36–3.88) than in the medium sleep duration group, whereas no such association was observed in men (46). A study from China reported that prolonged sleep duration (≥9 h) is correlated with frailty in men, whereas short sleep duration (<6 h) is related to frailty in women, indicating a gender-specific association (47). These inconsistencies may stem from differences in frailty measurement, methods of assessing sleep duration (self-report vs. actigraphy), or classifications of sleep duration (short sleep duration defined as <5 h, <6 h, or < 7 h) (39, 48). The ambiguous results of these studies also raise the question of whether older adults in various countries or regions have different sleep requirements (49). This study found no interaction among age, gender, and sleep duration. Previous research on populations under 60 years of age identified a significant interaction between sleep and gender, indicating that sleep exerts a protective effect on men and women. Therefore, the influence of gender on frailty must be assessed. Gender variations must be considered when formulating future preventive frailty intervention strategies.

Age contributes to frailty, and our research confirmed that frailty is a syndrome related to age. This study found that older men were more likely to become frail than older women. Prior studies indicate that women have a longer lifespan than men, but they frequently exhibit greater frailty than men of the same age (50). After women reach menopause, diminished estrogen levels can lead to a deficiency in vitamin D, adversely affecting neuromuscular balance and muscle strength, thereby increasing susceptibility to physical frailty (3). However, some studies revealed gender differences in the influence of frailty on adverse health outcomes, with males experiencing greater negative effects than women, which was consistent with the paradox of health survival between genders (51). Previous research have demonstrated that comorbidity is a significant risk factor for frailty (52–54). This result was consistent with the findings of this study. A meta-analysis in 2019 on the occurrence of cognitive-related frailty found that 72% of frail individuals presented with multimorbidity (55). The relationship between smoking and frailty is unclear, but it may be due to multiple factors, as tobacco smoke comprises a mixture of numerous toxic chemicals and compounds that affect various tissues and organs (56). In addition, smoking has been proven to be correlated with various physical and mental ailments, including chronic obstructive pulmonary disease, cardiovascular disease, and stroke, which may result in a deterioration of physical and cognitive performance (56). Research from numerous countries consistently demonstrated a positive association between smoking and increased frailty (57, 58). This study also found that an increase in weekly physical activities is correlated with a reduction in the incidence of frailty among older adults. Previous literature supports the association between MVPA and reduced frailty, and physical exercise can protect or improve the function of many physiological systems that deteriorate during frailty (59). Social interaction refers to behaviors that meet social and interactive needs, including physical, cognitive, and social activities (55). Engaging in social activities is an important aspect of social participation. Our research showed that individuals engaged in social activities exhibit a reduced likelihood of PF. The stress hypothesis posits that participating in social activities can reduce psychological stress and depression, lower cortisol levels, and enhance hippocampal neurons and physical function (60).

## Limitations

First, the measurement of sleep duration relies on self-reporting. Previous studies have demonstrated that individuals frequently overestimate their overall sleep time when self-reporting. Future research must employ objective tools to validate the association between sleep duration and frailty. Second, the cross-sectional study design inhibits our ability to determine causality. This design limitation means we can only reveal associations rather than infer causative relationships. Longitudinal studies are necessary to establish causality and better understand the temporal dynamics of sleep duration and frailty. Third, the data used for analysis are existing secondary data from CHARLS, not data specifically collected for the study of sleep and frailty. The results of this study are based on a nationally representative sample of community-dwelling older adults, so they may not be applicable to older adults in clinical settings. Despite these limitations, our research results are meaningful because they reveal a significant association between sleep duration and frailty in older adults. In the future, we will gather the latest data after COVID-19 and develop a new longitudinal data model to better investigate these relationships.

## Conclusion

Our findings indicate that, after adjusting for potential confounders, the duration of napping in older adults is not significantly associated with the likelihood of frailty. Furthermore, a U-shaped relationship exists between nighttime sleep duration and the probability of frailty, with optimal health benefits occurring around 7.5 h of sleep. Therefore, maintaining a nighttime sleep duration of 6–8 h is crucial for the health and well-being of older adults.

Policy makers should highlight the importance of adequate nighttime sleep among older adults. Public health initiatives can educate communities about healthy sleep practices and the dangers linked to inadequate and excessive sleep. Additionally, community and family healthcare providers should offer personalized sleep guidance and assistance to help older adults in establishing consistent sleep routines.

Healthcare professionals should incorporate sleep assessments as a critical component of health management for older adults. Regular monitoring and prompt treatment for sleep disorders can mitigate the prevalence of frailty. Individuals with sleep problems, tailored treatment plans, including behavioral therapy and medication, can improve sleep quality and overall health.

Further research should investigate the association between sleep and other health indicators in older adults to establish a thorough basis for improving the health of this demographic. Through continuous research and policy implementation, we may effectively reduce frailty risks and enhance the quality of life for older adults.

## Data Availability

The datasets presented in this study can be found in online repositories. The names of the repository/repositories and accession number(s) can be found below: here is a link to the data, an open database: https://charls.charlsdata.com/pages/data/111/zh-cn.html, if not available, please contact the corresponding author directly.
